# Gadolinium Protects *Arabidopsis thaliana* against *Botrytis cinerea* through the Activation of JA/ET-Induced Defense Responses

**DOI:** 10.3390/ijms22094938

**Published:** 2021-05-06

**Authors:** Juliana Santos Batista-Oliveira, Damien Formey, Martha Torres, Wendy Aragón, Yordan Jhovani Romero-Contreras, Israel Maruri-López, Alexandre Tromas, Kátia Regina Freitas Schwan-Estrada, Mario Serrano

**Affiliations:** 1 Centro de Ciencias Genómicas, Universidad Nacional Autónoma de México, Av. Universidad 2001, Cuernavaca, Morelos 62209, Mexico; julianaglomer@hotmail.com (J.S.B.-O.); formey@ccg.unam.mx (D.F.); mctorres@ccg.unam.mx (M.T.); waragon@ccg.unam.mx (W.A.); jhroco@ccg.unam.mx (Y.J.R.-C.); ismaruri@ccg.unam.mx (I.M.-L.); alexandre.tromas@gmail.com (A.T.); 2 Departamento de Agronomia, Universidade Estadual de Maringá, Maringá 87020, Brazil; krfsestrada@uem.br

**Keywords:** *Arabidopsis thaliana*, biostimulant, *Botrytis cinerea*, defense responses, gadolinium, rare-earth elements

## Abstract

Plant food production is severely affected by fungi; to cope with this problem, farmers use synthetic fungicides. However, the need to reduce fungicide application has led to a search for alternatives, such as biostimulants. Rare-earth elements (REEs) are widely used as biostimulants, but their mode of action and their potential as an alternative to synthetic fungicides have not been fully studied. Here, the biostimulant effect of gadolinium (Gd) is explored using the plant-pathosystem *Arabidopsis thaliana*–*Botrytis cinerea.* We determine that Gd induces local, systemic, and long-lasting plant defense responses to *B. cinerea*, without affecting fungal development. The physiological changes induced by Gd have been related to its structural resemblance to calcium. However, our results show that the calcium-induced defense response is not sufficient to protect plants against *B. cinerea*, compared to Gd. Furthermore, a genome-wide transcriptomic analysis shows that Gd induces plant defenses and modifies early and late defense responses. However, the resistance to *B. cinerea* is dependent on JA/ET-induced responses. These data support the conclusion that Gd can be used as a biocontrol agent for *B. cinerea*. These results are a valuable tool to uncover the molecular mechanisms induced by REEs.

## 1. Introduction

Two major factors determine the quantity and quality of plant-derived food: growth and development in the field and postharvest handling and storage conditions. Once a product is harvested, damage induced by microorganisms can cause 25% to 50% of the food to be lost [1]. Fungi of the genera *Alternaria, Aspergillus, Botrytis, Fusarium, Geotrichum, Gloeosporium, Penicillium, Mucor*, and *Rhizopus* are responsible for most of these losses [2,3]. In particular, the ubiquitous necrotrophic fungus *Botrytis cinerea* (Pers) has been shown to infect more than 200 plant species and to be responsible for “botrytis bunch rot” or “grey mold” symptoms. Due to these characteristics, *B. cinerea* has been classified as the second most important phytopathogen in existence [3]. To reduce the damage caused by *B. cinerea* and other fungi, farmers use different chemicals, including fungicides and biostimulants. The latter have been proposed as a new eco-friendly alternative to synthetic fungicides and are defined as naturally occurring molecules, elicitors, or microorganisms that enhance plant development, abiotic and biotic stress resistance, and/or crop quality traits [4,5].

Biostimulants that activate plant defense responses include polypeptides; glycoproteins; lipids; proteins; glycolipids; oligosaccharides; and microbe-, herbivore-, and damage-associated molecular patterns [4,5,6]. Defense responses primed by biostimulants include the activation of the plant innate immunity and the late defense responses. As part of the immune response, the accumulation of reactive oxygen species (ROS), calcium (Ca^2+^) influx, protein phosphorylation, mitogen-activated protein kinase (MAPK) signaling, and transcriptional induction of the early defense response genes take place [7,8,9]. After the initial response, the secondary defense responses are activated, including the production of histological barriers such as callose or lignin and the induction of salicylic acid—(SA), jasmonic acid—(JA), and ethylene—(ET) dependent signaling pathways [10]. These pathways finally lead to the induction of systemic acquired resistance (SAR) in non-infected distal organs of the plant [6,11,12]. The combined effect of these defense responses can efficiently stop disease induced by non-adapted pathogens, including fungi.

Rare-earth elements (REEs) are trace metals from the lanthanide group. While REEs are not known to be nutritionally essential to plants, they have been used as biostimulants in agriculture, particularly in China, to improve plant growth and development [13,14,15,16]. Depending on their concentration, REEs can have positive and negative effects on plant growth, development, and production (see reviews [17,18]). For instance, the application of a low concentration of neodymium (1, 3 or 5 mg/L) improved the germination rates of *Cassia obtusifolia* seeds, while higher concentrations inhibited them [19]. On the other hand, the application of 6 mg/L of neodymium increased the germination rates of *Astragalus membranaceu* seeds up to 42% [18], suggesting that the beneficial effects of REEs depend not only on the concentration but also on the plant species. Nevertheless, the beneficial effects of REEs have been related to their structural chemical resemblance to the secondary messenger, calcium [20]. Many REEs can displace the divalent Ca cation due to their trivalent charges and thus higher charge density, modifying an increased number of Ca-mediated biological processes [21]. In particular, calcium channels have been reported to be specifically blocked by the so-called “super calcium molecule” gadolinium, modifying multiple plant responses to abiotic and biotic stimuli [22,23]. Nevertheless, the effect of REEs on plant biology still remains mostly unknown [17]. For this reason, it is still not clear if the biological effects of REEs are only due to their structural chemical resemblance to Ca or to as-yet unknown mechanisms.

Nowadays, food production is based on the application of fertilizers—for instance, 18.8 × 10^10^ Kg of phosphate-based fertilizers are applied every year worldwide [24]. REEs are found as minor components of the raw material of phosphate-based fertilizers (monazites) [25,26]. For this reason, REEs are constantly applied to plants as biostimulants and/or as part of fertilizers, although basic information about them, such as their molecular mechanisms and the transcriptional changes induced by their application, are mostly unknown. On the other hand, their possible use as an alternative to synthetic fungicides has not been fully explored either. Only a handful of reports have studied the effect of REEs during plant–microbe interactions, some with contradictory outcomes. Some of these studies have indicated that the application of REEs induces defense responses against brown blast disease in rubber trees and fusarium wilt in tomato [27,28], while another report showed their inhibition of plant defense mechanisms [29]. Nevertheless, in order to become an alternative to synthetic fungicides, it is first necessary to properly characterize the molecular changes induced by REEs and then optimize their application and activity in the field.

In this report, we showed that the REE gadolinium (Gd) is a biostimulant that improves the growth and development of *A. thaliana* roots. Additionally, we determined that Gd protects plants against the necrotrophic fungus *B. cinerea*, without affecting the development of the fungus itself. Gd has a dose-dependent and long-lasting effect, triggering local and systemic defense responses against *B. cinerea*. To our knowledge, this is the first report of change in the plant transcriptome induced by Gd during the interaction with *B. cinerea* using genome-wide analysis (RNA-seq). This information will be a valuable tool to uncover the molecular mechanisms induced by REEs and will help in future efforts to improve and fully exploit their use in agriculture.

## 2. Results

### 2.1. Gadolinium Improves Root Development of A. thaliana

**Figure 1:**
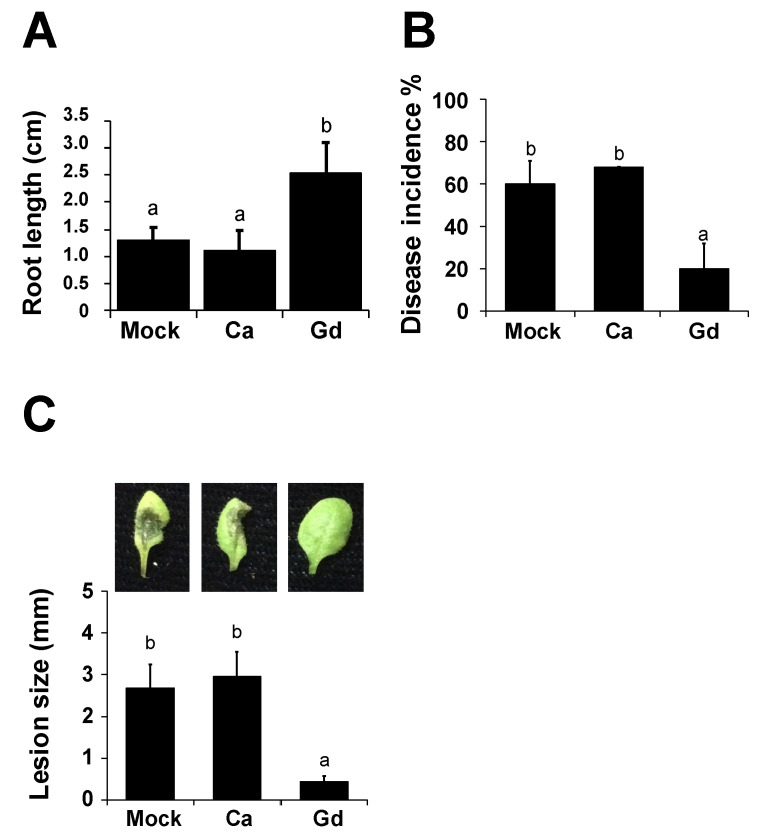
Gd improved the root development of *A. thaliana* and induced protection against *B. cinerea*. *A. thaliana* seeds were incubated for 1 h with distilled sterile water (mock), 0.2 g/L Ca, or Gd and germinated under in vitro conditions. Ten days post germination, the primary root length was measured (**A**). Four-weeks-old *A. thaliana* plants were sprayed until saturation with distilled sterile water (mock), 0.2 g/L Ca, or Gd (24 hpt). After this, 3 μL droplets containing a *B. cinerea* spore suspension (5 × 10^4^ spores mL^−1^) were applied and the 72 hpi disease incidence (**B**) and lesion size (**C**) were evaluated. A representative picture is included above each histogram as a visual illustration. Bars represent mean values (±SD) of three independent experiments (*n* = 30 for each experiment). Different letters above each bar represent statistically significant differences according to the Scott–Knott test (*p* < 0.05).

Our previous results indicated that the application of 0.2 g/L of gadolinium (III) nitrate hexahydrate (Gd(NO_3_)_3_·6H_2_O) has a positive effect on the development of *Glycine max* compared to plants treated with 0.2 g/L of calcium nitrate (Ca(NO_3_)_2_), henceforth identified as Gd and Ca [30]. Based on this observation and in order to better understand the molecular mechanisms induced by the exogenous application of gadolinium, we analyzed its effect on the model plant *A. thaliana*. *A. thaliana* seeds were germinated in the presence of distilled sterile water (as mock), 0.2 g/L Ca, or 0.2 g/L Gd. Ca was also used as a control because of its structural resemblance to Gd. Ten days after germination, a significant increase in root length was observed in Gd-treated plants compared to mock- and Ca-treated samples (Figure 1A). Afterwards, the same seedlings were transplanted to soil and the number of leaves was counted 30 days later. Once more, we observed a slight increase in the number of leaves in Gd-induced samples compared to the mock- and Ca-treated samples. However, these differences were not statistically significant (Figure S1). Additionally, no changes in germination rate or fresh or dry weight were observed between the mock-, Ca-, and Gd-treated samples (Figure S1). Taken together, these results suggest that Ca did not affect *A. thaliana* growth and that exogenous Gd application improved the root growth of *A. thaliana*.

### 2.2. Gadolinium Protects Arabidopsis thaliana Plants against the Necrotrophic Pathogen B. cinerea

Our next step was to determine if Gd has an effect in the well-characterized plant-pathosystem *A. thaliana–B. cinerea*. Four-week-old *A. thaliana* plants were pre-treated for 24 h (hpt) by spraying with 0.2 g/L Gd or Ca and distilled sterile water (as mock) and afterwards infected with *B. cinerea* spores. First, we measured the percentage of plants showing disease symptoms 72 h post infection (hpi), expressed as disease incidence (Figure 1B). No statistical differences were observed between the mock- and Ca-treated plants, suggesting that Ca did not affect the *A. thaliana–B. cinerea* interaction. However, we observed a 50% reduction in incidence in Gd-treated plants compared to Ca-treated control samples (Figure 1B). Additionally, when we characterized the disease severity by measuring the lesion size at 72 hpi, plants treated with Gd showed a strong reduction in the infection (almost to 80%) compared to the control Ca-treated samples (Figure 1C). Taken together, these results indicate that the exogenous application of Gd causes a strong reduction in disease incidence and infection severity from the necrotrophic pathogen *B. cinerea*. Additionally, since we determined that Ca did not affect the *A. thaliana–B. cinerea* interaction compared to H_2_O-treated plants (Figure 1B,C) and in order to eliminate the possible phenotypes induced by the structural resemblance between Ca and Gd, in the rest of the experiments we decided to use only Ca as a mock control.

### 2.3. Gd Does Not Affect the Development of B. cinerea

**Figure 2:**
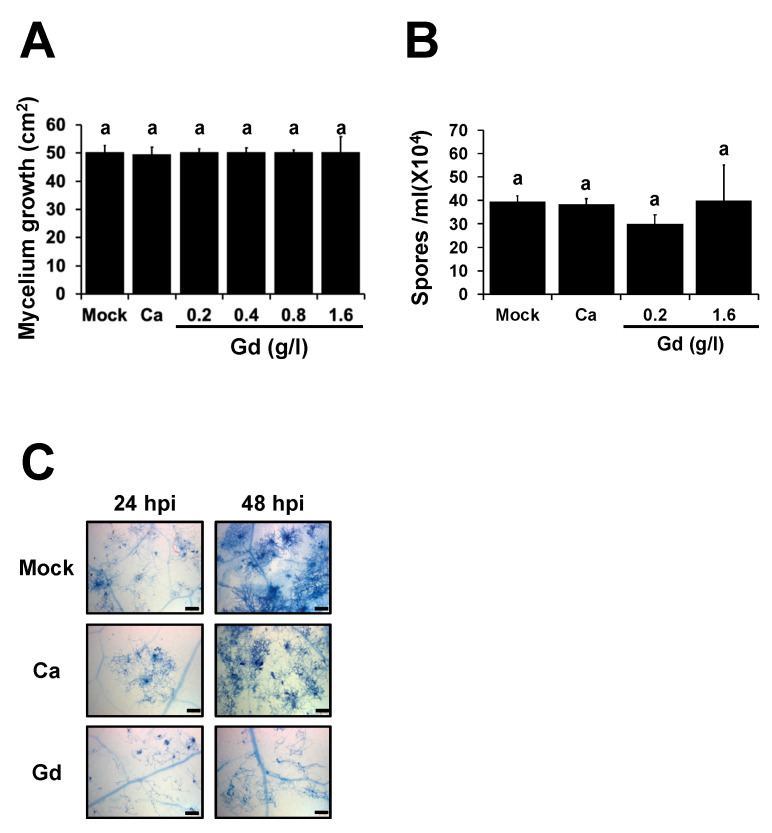
Gd does not affect the growth and development of *B. cinerea*. (**A**) A total of 5 μL of spore suspension of *B. cinerea* (5 × 10^4^ spores mL^−1^) was placed on the center of the Petri dish containing PDA supplemented with water (mock), 0.2 g/L Ca, and the indicated concentrations of Gd and incubated at 22 °C. Growth inhibition was evaluated by measuring the diameter of the mycelium on the dish 3 days post inoculation. (**B**) Spores produced by *B. cinerea* 10 days after growth on a Petri dish containing PDA supplemented with mock, 0.2 g/L Ca, and the indicated concentrations of Gd were isolated and quantified as previously described [33]. (**C**) *B. cinerea* development over *A. thaliana*-infected leaves was determined by trypan blue staining at 24 and 48 hpi. Representative images were selected as a visual illustration from two independent experiments. Bars represent mean values (±SD) of three independent experiments. Scale bar = 200 μm. Different letters above each bar represent statistically significant differences according to the Scott–Knott test (*p* < 0.05).

REEs have been shown to inhibit the development of several microorganisms [31,32]. To determine if the protective effect observed in plants (Figure 1C) was triggered either by the direct effect of Gd localized on the leaf surface or by the induction of the plant defense responses, we characterized the development of the pathogen in the presence of Gd under in vitro and in planta conditions (Figure 2). Spore suspensions, at a final concentration of 5 × 10^4^ spores per ml, were grown on a Petri dish containing PDA media supplemented with water (mock); 0.2 g/L Ca; or Gd at 0.2, 0.4, 0.8, and 1.6 g/L and incubated under optimal growth conditions for 10 days. We determined that Gd did not inhibit the growth of *B. cinerea* mycelium at any of the treatments (Figure 2A). Additionally, to determine if Gd has an effect on the development of *B. cinerea*, the number of spores was quantified. Once more, no statistical differences were observed at either of the two concentrations (0.2 and 1.6 g/L Gd) nor at 0.2 g/L Ca, compared to the mock-treated samples (Figure 2B). To further characterize these observations, the fungal growth in planta was followed by trypan blue staining. At 24 hpi, no differences in the hyphal development between mock-, Ca-, and Gd-treated leaves were observed. However, at 48 hpi the hyphal growth of *B. cinerea* on Gd-induced leaves was inhibited (Figure 2C). This suggests that the gemination of the spores was not modified, and while the initial compatible interaction between *B. cinerea* and *A. thaliana* might not be affected by Gd application, the progression of the infection is inhibited. Taken together, these results suggest that Gd induces a protective effect, as previously observed in planta (Figure 1), which could be mediated by the modifications of the plant defense responses rather than a direct effect on fungal growth and development.

### 2.4. Gd-Induced Protection against B. cinerea Is Dose-Dependent and Long-Lasting

**Figure 3:**
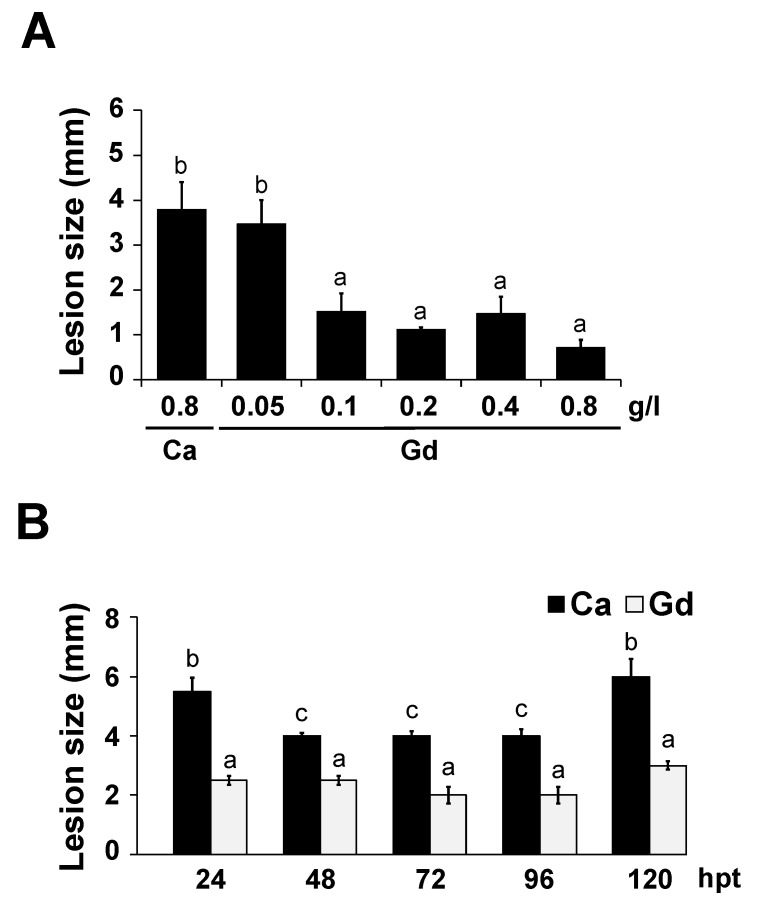
Gd-induced protection against *B. cinerea* is dose-dependent and long-lasting. (**A**) Four-week-old *A. thaliana* plants were pre-treated for 24 h (24 hpt) with the indicated Gd concentration or 0.8 g/L of Ca. After this, 3 µL droplets containing a *B. cinerea* spore suspension (5 × 10^4^ spores mL^−1^) were applied and infection symptoms were evaluated at 72 hpi by measuring lesion size. (**B**) Four-week-old *A. thaliana* plants were sprayed until saturation with 0.2 g/L of Ca or Gd for 24, 48, 72, 96, or 120 (hpt); after these times, 3 µL droplets containing *B. cinerea* spore suspension (5 × 10^4^ spores mL^−1^) were applied and infection symptoms were evaluated at 72 hpi by measuring lesion size. Bars represent mean values (±SD) of three independent experiments (*n* = 30 for each experiment). Different letters above each bar represent statistically significant differences according to the Scott–Knott test (*p* < 0.05).

To determine the optimal concentration of Gd-induced resistance against *B. cinerea*, a dose-dependent experiment was performed. We inoculated 4-week-old *A. thaliana* plants pre-treated for 24 hpt with 0.05, 0.1, 0.2, 0.4, and 0.8 g/L Gd, as well as 0.8 g/L Ca as a control, with *B. cinerea* spores, and measured the lesion size at 72 hpi (Figure 3A). No protection against the pathogen was observed on plants treated with the lowest concentration of Gd (0.05 g/L, Figure 3A). However, samples pre-treated with 0.1 to 0.8 g/L of Gd showed a strong reduction in lesion size of approximately 70%, compared to Ca-treated control plants (Figure 3A). It is noteworthy that plants pre-treated with 1.6 g/L of Gd showed spontaneous lesions and, for this reason, were not included in the analysis. These results show that a reduction in lesion size through the exogenous application of Gd is dose-dependent, with a strong inhibition of disease already occurring at a concentration of 0.1 g/L of Gd.

To evaluate for how long Gd can protect *A. thaliana* plants against *B. cinerea*, different pre-treatment times were analyzed by measuring the lesion size at 72 hpi. Interestingly, the Ca-treated control plants showed a significant reduction in lesion size at 48, 72, and 96 hpt of approximately 30% compared to 24 and 120 hpt, suggesting that Ca has an effect on the plant–pathogen interaction at these time points (Figure 3B). Nevertheless, at all the time points analyzed, Gd-treated plants always showed a significant reduction in lesion size compared to their corresponding control Ca-treated samples, fluctuating between 60% (24 and 120 hpt) and 40% (48, 72, and 96 hpt) (Figure 3B). Thus, these results indicate that Gd exerts a protective effect against *B. cinerea* over several days.

### 2.5. Gd Triggers a Systemic Defense Response to B. cinerea

**Figure 4:**
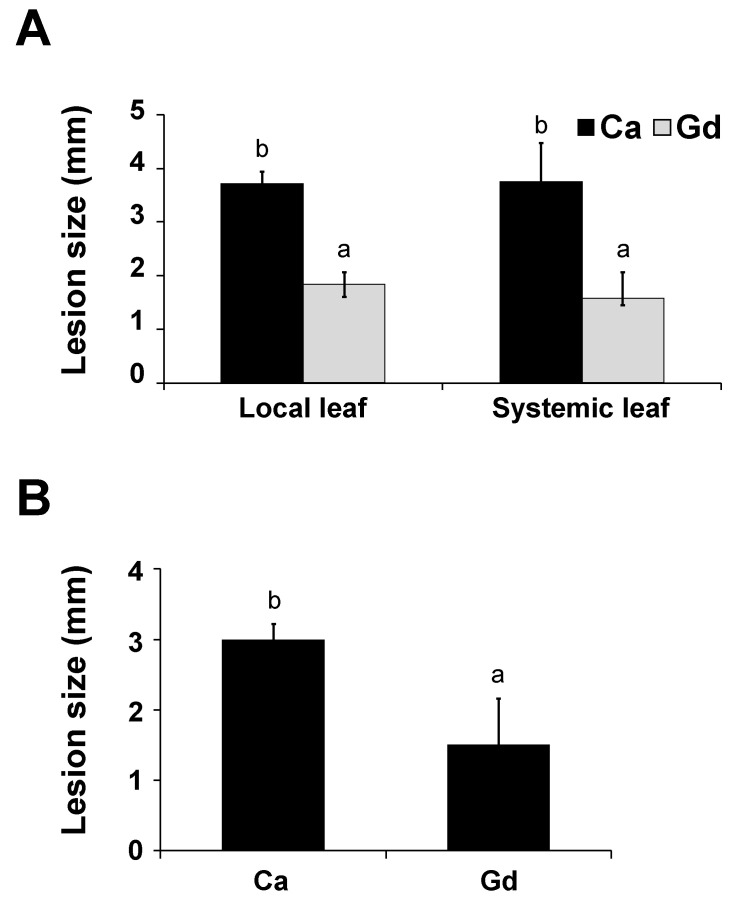
Systemic effect induced by Gd against *B. cinerea* in *A. thaliana* plants. Four-week-old *A. thaliana* plants were pre-treated as follows: (**A**) half of the rosette leaves were pre-treated with 0.2 g/L Gd or Ca (local) and the other half with H_2_O (systemic); (**B**) plants were watered until saturation with 0.2 g/L of Gd or Ca. At 24 hpt, 3 µL droplets containing *B. cinerea* spore suspension (5 × 10^4^ spores mL^−1^) were applied. Infection symptoms were evaluated at 72 hpi by measuring lesion size. Bars represent mean values (±SD) of three independent experiments (*n* = 30 for each experiment). Different letters above each bar represent statistically significant differences according to the Scott–Knott test (*p* < 0.05).

To further characterize the Gd-induced plant defenses against *B. cinerea*, we analyzed whether a systemic protection was activated (Figure 4). Firstly, half of the rosette leaves from 4-week-old *A. thaliana* plants were pre-treated with 0.2 g/L of Gd or Ca (local) and the other half with H_2_O (systemic). Then, at 24 hpt we inoculated all the leaves with *B. cinerea* spores and measured the lesion size at 72 hpi. Plants treated with Gd showed a strong reduction in the lesion sizes for both tissues (local and systemic) compared to their respective Ca-treated control leaves (Figure 4A). In another tray, 4-week-old *A. thaliana* plants were watered to saturation with a solution of 0.2 g/L of Gd or Ca and untreated leaves were inoculated for 24 hpt with *B. cinerea* and the lesion size measured at 72 hpi. Gd-watered plants showed an approximately 50% reduction in lesion size compared to the Ca-watered control plants (Figure 4B). These results indicate that Gd induces a systemic defense response against *B. cinerea* in *A. thaliana* plants.

### 2.6. Gd Does Not Modify the Cuticle Permeability but Triggers a ROS Burst

**Figure 5:**
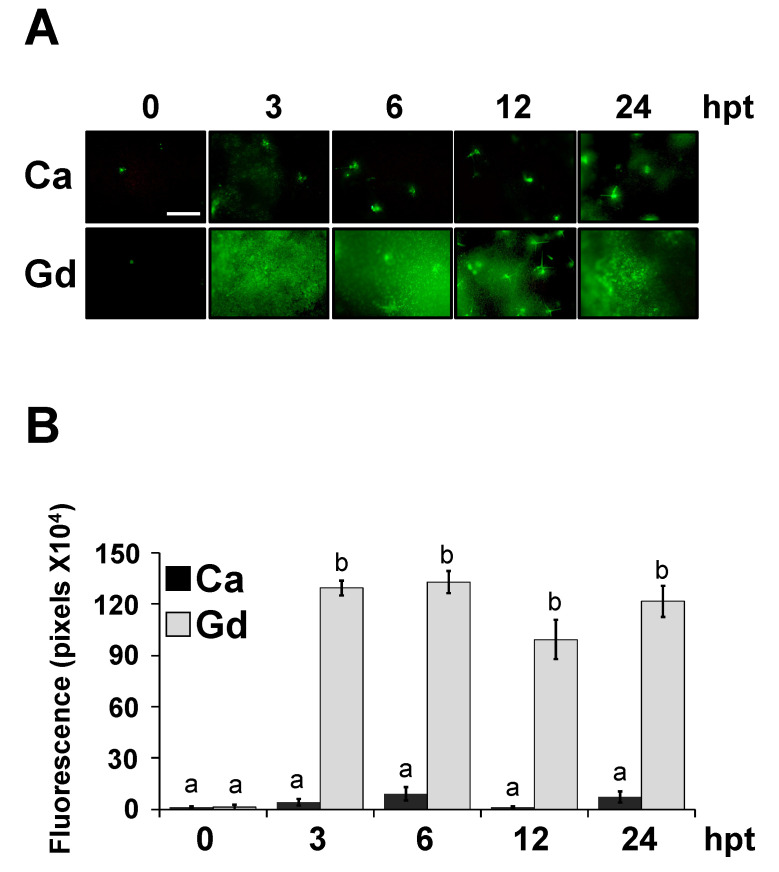
Gd-induced accumulation of ROS. (**A**) Leaves from 4-week-old *A. thaliana* plants treated with 0.2 g/L of Ca or Gd were stained using DCF-DA to detect basal ROS levels at the indicated time point (**B**) Densitometric quantification of ROS production at indicated time points after Ca or Gd treatment. Bars represent the mean values (±SD) of three independent experiments (*n* = 30). Different letters above each bar represent statistically significant differences according to the Scott–Knott test (*p* < 0.05). Scale bar = 200 µm.

We have described that cuticle-related mutants, as part of a defensive syndrome, showing an increase in leaf permeability and a rapid induction of the plant innate immunity, including the accumulation of reactive oxygen species (ROS) and resistance against *B. cinerea* [34,35]. In order to determine if the Gd-triggered defense responses against *B. cinerea* are mediated by a similar syndrome, we quantified the leaf permeability using three different methods: measuring the efflux of chlorophyll, toluidine blue staining, and calcofluor staining, as previously described [33]. Four-week-old *A. thaliana* plants were treated for 24 h with 0.2 g/L of Gd or Ca. Leaf permeability was not modified by exogenous Gd application, since no differences were observed between Ca- and Gd-treated samples in any of the methods used (Figure S2). Then, using a similar treatment, we quantified the presence of ROS at 0, 3, 6, 12, and 24 hpt (Figure 5) through histological analysis and densitometric quantification with the probe 5-(and-6)-carboxy-2,7-dichlorodihydrofluorescein diacetate (DCF-DA), which detects a broad spectrum of ROS. Histological analysis showed that, at 3 hpt, a strong accumulation of ROS induced by Gd (Figure 5A) was already detected and further maintained without significant changes up to 24 hpt, while with Ca-treated plants ROS was not induced at any of the time points (Figure 5A). These observations were confirmed by densitometric quantification (Figure 5B). Taken together, these results suggest that the ROS burst detected after Gd application was induced directly by Gd treatment rather than by a modification of the cuticle permeability.

### 2.7. Gadolinium Up-Regulates the Responses to Biotic Stimulus and Represses the Responses to Abiotic Stimulus

**Figure 6:**
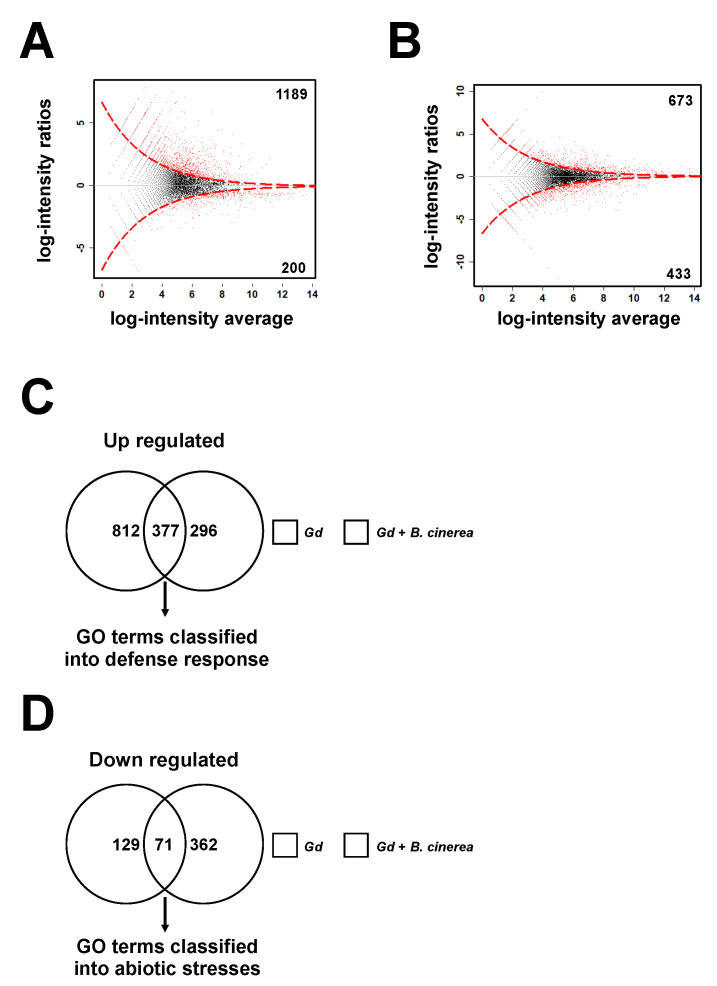
RNAseq analysis of Gd-induced *A. thaliana* plants. Five 4-week-old *A. thaliana* plants were sprayed until saturation with 0.2 g/L of Ca or Gd (24 hpt) and then infected with *B. cinerea* (24 hpi). Total RNA for each condition, from three independent experiments, was pooled and sequenced (RNA-seq). (**A**) MA plot of Gd- versus Ca-treated samples at 24 hpt. (**B**) MA plot of Gd- versus Ca-treated samples infected with *B. cinerea* at 24 hpi. The red points represent DEGs (*p*-value < 0.05), while black dots indicate genes with a similar expression. The dotted red line shows the limit between similarly and differentially expressed genes. The black horizontal line at zero provides a visual check for symmetry. Venn diagrams representing overlapping or non-overlapping gene sets of up-regulated DEGs (**C**) and down-regulated genes (**D**), respectively, were identified in *A. thaliana* plants induced with 0.2 g/L of Ca or Gd and infected with *B. cinerea*, as indicated.

To identify the genetic elements that participate in Gd-induced responses, we studied the transcriptional changes in the Gd-treated plants using a genome-wide RNA sequencing analysis (RNA-seq). Total RNA was isolated from 4-week-old *A. thaliana* plants at two different time points—first at 24 hpt with 0.2 g/L of Gd or Ca and then from plants at 24 hpi inoculated with *B. cinerea* (Figure 6). Gd-pretreated plants showed 1189 and 200 genes with significantly higher and lower expressions, respectively, compared to the Ca-induced ones (Figure 6A; Supplementary Data 1). Gd-treated plants that were infected with *B. cinerea* showed 673 and 433 genes with higher or lower expressions, respectively, compared to Ca-treated plants (Figure 6B; Supplementary Data 2). These results suggest that the application of exogenous Gd modifies the transcriptome to improve the growth and defense responses of *A. thaliana*.

From the DEGs detected in the Gd application conditions compared to Ca (Table S1), we wanted to identify those that had higher and lower expressions after the infection by *B. cinerea* took place. A total of 377 induced genes and 71 repressed genes follow this pattern (Figure 6C,D). Interestingly, the GO analysis of these DEGs revealed that almost all the genes with a higher expression were classified into the defense response mechanisms, including responses to fungi and innate immune responses (Table S1). In contrast, the repressed genes were classified mostly in the category of responses to abiotic stresses (Table S1). These results suggest that different sets of mechanisms are induced or repressed by Gd application.

### 2.8. Plant Defense Response Genes Are Induced by Gadolinium Treatment and during the Interaction with B. cinerea

**Figure 7:**
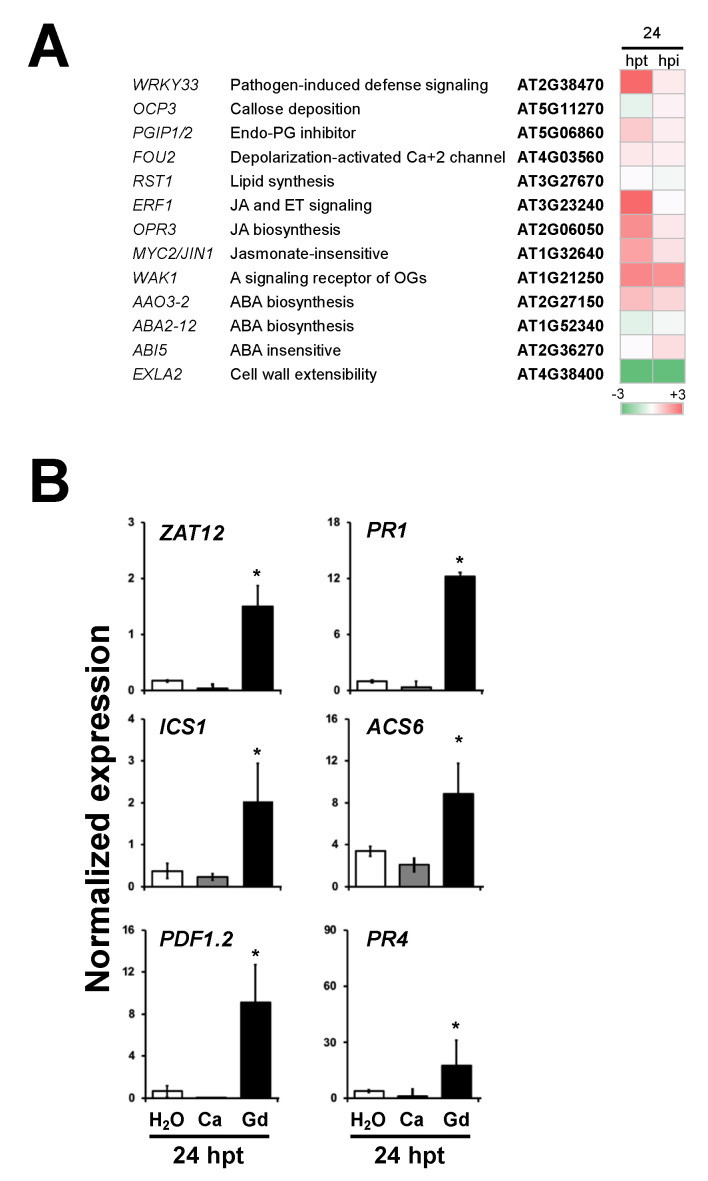
Gd transcriptionally activate the plant defense response genes. (**A**) Heatmaps of the expression log2-fold changes of genes previously described to be involved in the resistance against *B. cinerea* [10], compared to the corresponding controls, from RNAseq data at 24 hpt and hpi. (**B**) Four-week-old *A. thaliana* plants pretreated with H_2_O (mock), 0.2 g/L Ca, or Gd (24 hpt). Quantitative real-time PCR (qRT-PCR) expression analysis of *ZAT12*, *ICS*1, *PR1*, *ACS6*, *PDF1.2,* and *PR4* was determined and normalized with respect to the mean of two reference genes *AT4G26410* and *AT1G72150*, as previously described [43,44]. A representative experiment with three technical replicates is shown (±SE). Three independent experiments were carried out with similar results. Asterisk above each bar represents statistically significant differences to the mock-treated samples according to the Student’s *T*-test (*p* < 0.05).

The transcriptional activation of the defense responses induced by Gd was analyzed using the bioinformatics tool MapMan [36]. From this analysis, an induction of the responses to biotic stress was observed in Gd-pretreated plants (24 hpt) (Figure S3A) and Gd-induced plants later infected with *B. cinerea* (24 hpi) (Figure S3B). A list of candidate genes that have been characterized as improving the resistance against *B. cinerea* was recently described [10]. We analyzed the accumulation of these candidate transcripts in Gd-induced plants (24 hpt) and the plants after the interaction with the pathogen (24 hpi) in comparation to Ca-treated samples (Figure 7A). Eight out of 13 genes were induced at 24 hpt, while 10 were up-regulated at 24 hpi, suggesting that Gd transcriptionally induces defense responses against *B. cinerea*. To further confirm and validate the genome-wide RNA sequencing analysis (Supplementary Data 1 and 2), we further characterized by qRT-PCR the accumulation of transcripts of representative *A. thaliana* genes previously described to be involved in the plant–microbe interactions from the ROS—(*ZAT12*) [37], SA—(*ICS1* and *PR1*) [38,39], JA—(*PDF1.2*) [40], and ET-signaling pathways (*ACS6* and *PR4*) [41,42]. This was conducted in 4-week-old *A. thaliana* plants pretreated with H_2_0 (mock), Ca, or Gd (24 hpt) (Figure 7B). Remarkably, we determined that the mock- and Ca-treated samples showed similar expression patterns for all the genes, confirming the validity of the Ca control. On the other hand, we observed that at 24 hpt, all the analyzed genes were induced in Gd-treated plants compared to the Ca-induced ones. It is worth mentioning that for all the genes analyzed, the expression signatures were similar to those detected by RNAseq (highlighted in Supplementary Data 1). Additionally, based on the structural similarities between Gd and Ca, one might expect that Ca-induced genes will be also up-regulated by Gd. In agreement with this, several Ca-responsive genes were part of the Gd-induced ones, such as the calcium-binding EF-hand family protein (AT3G01830) and the vacuolar calcium-binding protein-related (AT1G62480) (Supplementary Data 1), validating our analysis. Taken together, these results suggest that application of Gd transcriptionally activates the selected ROS-, SA-, JA-, and ET-induced defense response genes that were previously identified as part of the plant defense mechanisms against *B. cinerea* [33,43].

### 2.9. Gd-Induced Defense Response Against B. cinerea Is Dependent of JA and ET

**Figure 8:**
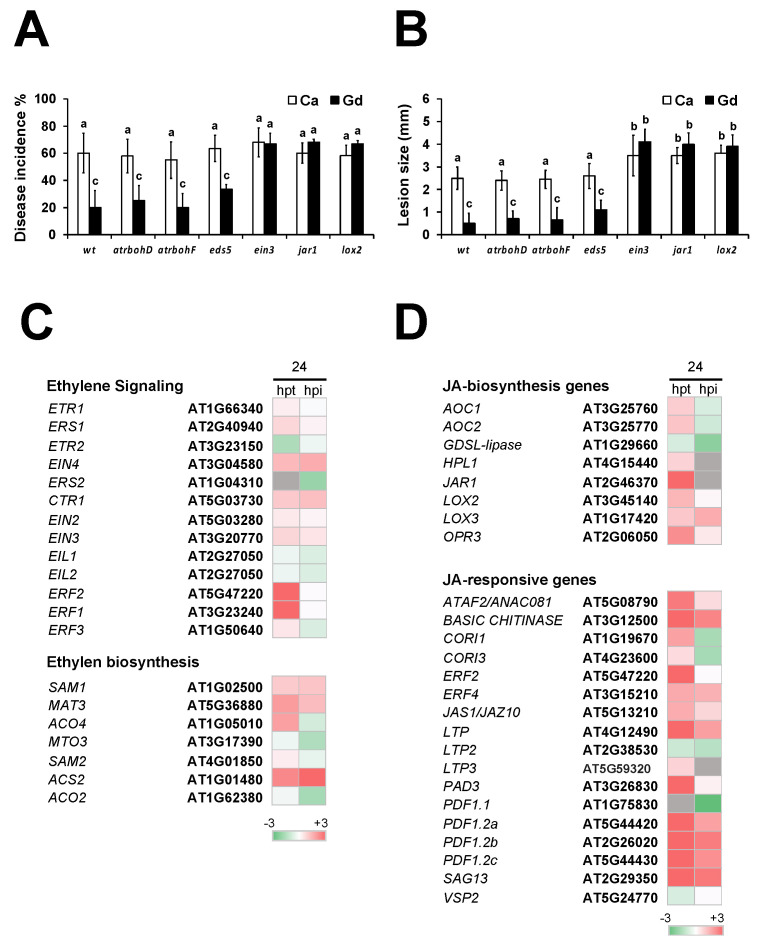
Role of ROS, SA, and JA in Gd-induced resistance to *B. cinerea* in *A. thaliana*. The *AtrbohD, AtrbohF, eds5, ein3, jar1,* and *lox2* mutants were evaluated. (**A**) Four-week-old *A. thaliana* plants were sprayed until saturation with 0.2 g/L of Ca or Gd. After 24 hpt, 3 µL droplets containing a *B. cinerea* spore suspension (5 × 10^4^ spores mL^−1^) were applied and disease incidence was determined by measuring the percentage of plants showing disease symptoms at 72 hpi. (**B**) The disease severity was determined by measuring the lesion size of all the infected leaves. Heatmaps of the expression of log2-fold changes in ET- (**C**) and JA-related genes (**D**), compared to the corresponding controls, from RNAseq data at 24 hpt and hpi. Bars represent mean values (±SD) of three independent experiments (*n* = 50). Different letters above each bar represent statistically significant differences according to the Scott–Knott test (*p* < 0.05).

As part of the defense response against *B. cinerea*, the plant transcriptome is modified, including the activation of ROS-, SA-, ET-, ABA-, JA-, and ET-induced signaling pathways [45]. In this work, we show that Gd transcriptionally induces ROS-, SA-, JA-, and ET-induced defense response genes (Figure 7). In order to determine the effect of each response pathway on Gd-triggered defenses, we analyzed mutants impaired in ROS, SA, JA, and ET accumulation. Measuring the disease incidence and lesion size, we determined that plants were still protected against *B. cinerea* after Gd application in the mutants of *NADPH oxidase D* (*AtrbohD*) and *F* (*AtrbohF*) involved in ROS production (Figure 8A,B). On the other hand, even the SA-deficient mutant *eds5* showed a slight increase in disease incidence and lesion size after Gd application, although these changes were not statistically significant compared to those in Col-0 wt-treated plants (Figure 8A,B). However, for the ET- and JA-related mutants *ethylene-insensitive* 3 (*ein3*), *jasmonate-amido synthetase* (*jar1*), and *lipoxygenase 2* (*lox2*), we observed a similar disease incidence and lesion sizes in Gd-induced plants compared to the Ca-treated samples, suggesting that the Gd-induced defense response is mediated by JA and ET (Figure 8A,B). To confirm this observation, we selected the genes from our transcriptomic analysis that had been previously described to be involved in ET- and JA-related responses that have also been characterized to be involved in resistance to *B. cinerea* [46], and we analyzed their differential accumulation status (Figure 8C,D). For ET-related genes, 60% were induced at 24 hpt with Gd, while 30% were up-regulated after the interaction with the pathogen took place (24 hpi, Figure 8C). On the other hand, for JA-related genes 78% were induced after the treatment with Gd (24hpt) and 48% kept this expression pattern after the interaction with *B. cinerea* (24 hpi) (Figure 8D). Taken together, these results indicated that the Gd-triggered defense response against *B. cinerea* is mostly dependent on the JA/ET-induced responses.

## 3. Discussion

### 3.1. A Transcriptome Analysis Provides New Insights into the Molecular Mechanisms Involved in the Gd Response in A. thaliana

During the last 30 years, REEs have been widely used as biostimulants in the farmlands of China and other countries [47,48]. Previous physiological analyses indicated that the exogenous application of REEs modifies calcium-induced responses, such as the structure and function of the cytoplasmic membranes, photosynthesis, the modulation of hormone metabolism, and the increased efficiency of water use [48]. Nevertheless, their modes of action(s), in particular at the transcriptional level and during biotic interactions, are still poorly understood [49]. In this report, we show that *A. thaliana* treated with gadolinium (Gd) protected the plants against *B. cinerea* (Figure 1). In order to uncover the molecular mechanisms behind this effect, we performed a genome-wide transcriptional analysis (Supplementary Data 1). The GO analysis revealed that almost all the induced genes were classified into the defense response mechanisms, while the repressed ones were classified mostly in response to abiotic stresses (Figure 6). Interestingly, several processes which had been previously associated with changes in root growth were identified among the repressed DEGs, including responses to abscisic acid (ABA) and water deprivation. High concentrations of ABA have been shown to inhibit root growth [50], while water deficiency stimulates it [51]. These reports support the view that the information generated in this work can be used as a starting point to unravel the molecular mechanisms behind REE-induced responses, potentially leading to the optimization of their application as biostimulants.

### 3.2. Gd Is a Novel Biocontrol against the Broad Host-Range Necrotrophic Fungus B. cinerea

One of the limiting factors in food production is disease caused by fungi [52], with *B. cinerea* being one of the major ones responsible for these losses [2]. Most of the treatments to control the infections inflicted by this pathogen are based on the application of synthetic fungicides. However, an increase in worldwide regulatory policies and the claim to reduce their application due to the possible harmful side effects have led to searches for new eco-friendly alternatives, such as biostimulants. Despite the generalized use of REEs as biostimulants in agriculture, only a few reports have studied its impacts on plant–microbe interactions [27,28,29], and to our knowledge none have described the inhibition or modification of the interaction with *B. cinerea*. Here, we show that Gd can protect *A. thaliana* plants against *B. cinerea* by inducing defense responses. We also show that even at high Gd concentrations, the growth and development of *B. cinerea* is not affected by Gd or Ca under in vitro or in planta conditions (Figure 2). This is somewhat unexpected, since REEs have been described as inhibiting the development of other microorganisms, and high concentrations of CaCl_2_ can decrease the spore germination and mycelial growth of *B. cinerea* [53]. These results suggest that the protective effect induced by Gd is mediated by the modification of the plant defense mechanisms rather than a direct inhibitory effect on the pathogen. Additionally, we determined that the Gd-induced protection is dose-dependent and lasts for 5 days (Figure 3). All these results indicate that Gd has the potential to be used as a biocontrol against this agronomically important pathogen.

### 3.3. Gd Induces Protection against B. cinerea by Activating Early and Late Defense Responses, in Particular the JA- and ET-Induced Signaling Pathway

Our RNA-seq analysis shows that early and late defense responses are transcriptionally induced after the application of Gd (Table S1). As part of the early defense mechanisms, plants can trigger innate immunity, which includes the accumulation of reactive oxygen species (ROS), calcium (Ca^2+^) influx, protein phosphorylation, MAPK-dependent signaling cascades, and the transcriptional induction of defense response genes [7,8]. In this work, we observed a fast accumulation of intracellular ROS after Gd application (3 hpt), which was further maintained for 24 h (Figure 5). This is consistent with the fact that we have described a direct link between ROS accumulation and the resistance to *B. cinerea* in previous studies [33,34,54]. This could be sufficient to explain the reduction in infection observed in Gd-treated plants. However, it is necessary to mention that other REEs have been evidenced to modify ROS accumulation [55]. Additionally, we determined that mutants of the enzymes *AtrbohD* and *AtrbohF*, involved in intracellular ROS production, can still be protected after Gd treatment (Figure 8), suggesting that either only intracellular ROS is produced or that other genes are probably implicated in Gd-induced ROS production. It would be interesting to test this hypothesis using mutants deficient in extracellular ROS production. Based on this information, we cannot discard the idea that the ROS burst might be triggered specifically by Gd or as a general response to incubation with REEs, and can only assume that this is part of the defense mechanism against *B. cinerea*.

The activation of innate immunity is followed by secondary mechanisms, including the induction of SA-, JA-, and ET-dependent signaling pathways, finally leading to the activation of systemic acquired resistance (SAR) in non-infected distal parts of the plant [6,11,12]. We revealed that SA-, JA-, and ET-induced genes were transcriptionally activated after Gd treatment and during the interaction with *B. cinerea* (Figure 7 and Supplementary Data 1 and 2). However, we observed that the mutant impaired in SA accumulation (*eds5*) shows a slight, but not statistically significant, reduction in Gd-induced protection. The protection induced by this REE was completely lost in *ein3*, *jar1,* and *lox2* mutants, which are impaired in JA- and ET-induced responses (Figure 8). SA-triggered defense responses have been reported to be involved against necrotrophic pathogens in *A. thaliana* [56,57] and tobacco [58]. However, other studies have reported that *A. thaliana* defense responses against *B. cinerea* depend mostly on JA and ET [59,60,61]. Our results indicate that the protective effect of Gd against this pathogen depends on ET- and JA-dependent signaling pathways.

### 3.4. Ca might Elicit the Defense Responses but Not as Strongly as Gd

To cope with environmental stresses, plant responses are intimately coordinated by the complex signaling networks of the so called “trio signaling” messengers: ROS, electrical signals, and calcium [62]. In particular, Ca plays an important role in plant–pathogen interactions, since once an attack is perceived its intracellular concentration is rapidly increased, activating defense responses in a local and systemic fashion [63]. As mentioned, the physiological changes induced by REEs have been related to their structural chemical resemblance to calcium [20]. In agreement, Gd has been described to elicit a similar signaling pathway to this secondary messenger [64]. In this report, in order to reduce the expected phenotypes induced by the structural and functional resemblance of Gd and Ca, we used calcium nitrate as a control. Ca has been described to inhibit the spore germination and mycelial growth of *B. cinerea* [53]. Nevertheless, we did not detect a reduction in infection due to *B. cinerea* on *A. thaliana* plants treated with Ca (Figure 1B,C) either locally or systemically at this concentration (Figure 4). However, interestingly, during the time-course experiment, we observed a significant reduction in lesion size after 48, 72, and 96 hpt in Ca-induced samples, compared to at 24 hpt in Ca-treated plants (Figure 3B). This reduction, however, was never as strong as that measured in Gd-treated plants at any of the time points analyzed (Figure 3B). This result suggests that both Ca and Gd might elicit defense responses, though at different times, against the necrotrophic pathogen *B. cinerea*. A detailed analysis of the Ca-induced defense mechanisms after 48, 72, and 96 hpt and of all the DEGs identified in this work might help us to uncover and understand the potential differences between Ca- and Gd-induced defense pathways.

Finally, even if our initial goal was to characterize the effect of Gd on *B. cinerea*, it is worth mentioning that our RNA-seq analysis suggests that this REE might extend its effect over other biotic and abiotic stresses. Among the biological processes induced by Gd treatment, several defense responses were identified, including responses to other organisms, such as bacteria and response to salicylic acid (SA). For instance, the SA-induced signaling pathway is a central part of the plant defense responses to multiple pathogens, including infections by viruses, bacteria, and fungi [65]. On the other hand, our GO analysis revealed that repressed genes were classified mostly in response to abiotic stimuli, including responses to abscisic acid (ABA) and water deprivation. REEs have been described to increase plant resistance to different abiotic stresses [17,66]. With this in mind, our results seem to point towards a possible use of Gd in the context of sustainable agricultural production to enhance plant tolerance to numerous biotic and abiotic stresses. Nevertheless, to continue using Gd as a plant stimulator it will be necessary to take into account that long-term use might increase its potential as a source of pollution in the soil, as previously described for other REEs [67].

## 4. Materials and Methods

### 4.1. Plant Material and Growth Conditions

*Arabidopsis thaliana* (L.), Heynh ecotype Columbia-0 (Col-0), and *lox2* mutant were obtained from the Nottingham Arabidopsis Stock Centre (Nottingham, UK). The *A. thaliana* mutants used in this work (all in the Col-0 background) were the following: *AtrbohD*, *AtrbohF* [68], *eds5* [69] *jar1* [70], and *ein3* [71]. Plants grown under in vitro conditions were generated from surface-sterilized seeds germinated in square Petri dishes containing Murashige and Skoog (MS) media supplemented with 0.8% agar and 1% sucrose. Plates were kept at 4 °C for two days and incubated vertically at 21 °C for 10 days in a growth chamber with a 16 h light/8 h dark photoperiod. For ex vitro experiments, *A. thaliana* seeds were grown on a pasteurized (applying heat until the mix reaches 82 °C for 30 min) soil mix of humus/perlite (3:1), kept at 4 °C for two days, and then transferred to the growth chamber. Plants were grown for 4 weeks in a 12 h light/12 h dark cycle with a 60–70% relative humidity, a light intensity of 200 µmol/m^2^/s, a day temperature of 20–22 °C, and a night temperature of 16–18 °C. For in vitro experiments, surface-sterilized *A. thaliana* seeds were incubated for 1 h in distilled sterile water (mock), 0.2 g/L (0.0012 M) calcium nitrate (Ca(NO_3_)_2_), or 0.2 g/L (0.00044 M) gadolinium (III) nitrate hexahydrate (Gd(NO_3_)_3_·6H_2_O). Seeds were germinated as described above and the germination rate, primary root length, and fresh and dry weight were measured 10 days post germination.

### 4.2. Gadolinium Treatment and B. cinerea Plant Inoculation

Four-week-old *A. thaliana* plants were pre-treated by spraying to the point of run off with 0.2 g/L of gadolinium (III) nitrate hexahydrate, 0.2 g/L of calcium nitrate, or H_2_O for 24, 48, 72, 96, or 120 h post-treatment, hpt. After this time, 3 µL droplets of *B. cinerea* spore suspension (5 × 10^4^ spores mL^−1^) were applied. The procedures of *B. cinerea* infection, including disease incidence and measurement of lesion size, were determined 72 h post-infection (hpi), as previously described [33]. For the dose–response assay, plants were pre-treated for 24 h (24 hpt) with the indicated concentration of gadolinium (III) nitrate hexahydrate or calcium nitrate and then infected with *B. cinerea* and evaluated at 72 hpi.

### 4.3. Systemic Defense Response Analysis

For the systemic assay, two methodologies were used: (i) half of the rosette leaves of 4-week-old *A. thaliana* plants were pre-treated by spraying with 0.2 g/L of gadolinium(III) nitrate hexahydrate or calcium nitrate (local) and the other half with H_2_O (systemic) and (ii) 4-week-old *A. thaliana* plants were pre-treated (watering the soil until saturation) with 0.2 g/L of gadolinium(III) nitrate hexahydrate or calcium nitrate for 24 h (24 hpt). For both conditions, leaves were infected with *B. cinerea* and evaluated at 72 hpi, as described above.

### 4.4. In Planta B. cinerea Growth Analysis

Fungal hyphae were stained and plant cell death assayed as previously described [72]. Once stained, leaves were imbibed in 20% glycerol for 1 h and observed using a microscope with bright-field settings. Representative images were selected as a visual illustration.

### 4.5. In Vitro Inhibitory Assay of B. cinerea Growth

The *B. cinerea* strain BMM (originally isolated from grape wine) was provided by Brigitte Mauch-Mani (University of Neuchatel, Switzerland). *B. cinerea* in vitro growth and preparation of spore suspension were performed as previously described [33]. For the inhibition assay, 5 μL of a spore suspension of *B. cinerea* (5 × 10^4^ spores mL^−1^) was placed at the center of a Petri dish containing potato dextrose agar media (PDA) supplemented with 0.2 g/L of calcium nitrate or 0.2, 0.4, 0.8, or 1.6 g/L of gadolinium(III)nitrate hexahydrate, as indicated in the figure legends. The plates were incubated at 22 °C for 3 days and then the diameter of the mycelial growth was determined using Image J version 1.51 (U. S. National Institutes of Health, Bethesda, MD, USA), as previously described [73]. Ten days post incubation, *B. cinerea* spores were collected from the plates and re-suspended in 5 mL of H_2_O and quantified using a Neubauer cell counting chamber.

### 4.6. Cuticle Permeability Analysis

We recently described that cuticle-related mutants show an increase in leaf permeability and a rapid induction of plant innate immunity, including the accumulation of reactive oxygen species (ROS) [34,35]. To determine the cuticle permeability, three methods were used: (*i*) calcofluor white staining, (*ii*) toluidine blue staining, and (*iii*) chlorophyll extraction and quantification [33,34,54]. To stain with calcofluor white, leaves were bleached in absolute ethanol overnight, equilibrated in 0.2 M NaPO_4_ (pH 9) for 1 h, and incubated for 1 min in 0.5% calcofluor white in 0.2 M NaPO_4_ (pH 9). Leaves were rinsed in NaPO_4_ buffer to remove excess calcofluor white and were viewed under a UV light. Toluidine blue staining was carried out by placing 6 μL droplets on the leaf surface of 0.025% toluidine blue solution dissolved in ¼ PDB. After incubation for 2 h, the leaves were washed gently with distilled water to remove excess solution from them. Representative images were selected as a visual illustration. For chlorophyll extraction and quantification, leaves were weighed and immersed in 30 mL of 80% ethanol. Chlorophyll suspension was sampled in the dark at room temperature with gentle agitation at 2, 5, 10, 20, 30, 40, 50, 60, and 120 min after immersion. The chlorophyll content was determined by measuring the absorbance at 664 and 647 nm and the micromolar concentration of total chlorophyll per gram of fresh weight of tissue was calculated using the following equation: (7.93 × (A_664_ nm) + 19.53 × (A_647_ nm)) g^−1^ fresh weight.

### 4.7. Detection of ROS

ROS were detected using the fluorescent probe 5-(and-6)-carboxy-2,7-dichlorodihydrofluorescein diacetate (DCF-DA), as previously described [33]. Representative images were selected as a visual illustration.

### 4.8. RNA Extraction and Genome-Wide Transcriptomic Analysis

For the three independent experiments, leaves from 5 *A. thaliana* plants were harvested, pooled and immediately frozen in liquid nitrogen and kept at −80 °C. Two conditions were analyzed. The first was at 24 hpt with 0.2 g/L of gadolinium(III)nitrate hexahydrate or calcium nitrate and the second was from plants pre-treated by spraying the entire leaf with gadolinium(III)nitrate hexahydrate (24 hpt) or calcium nitrate and infected for 24 hpi with *B. cinerea* by spraying the spore suspension all over the leaf. Total RNA was extracted as described in the manufacturer’s protocols using the Spectrum^TM^ Plant total RNA Kit (Sigma Aldrich, San Luis, MO, USA). The integrity of the extracted RNA was measured by agarose gel electrophoresis (1.2%). The NanoDrop 2000/2000c (Thermo Fisher Scientific, Waltham, MA, USA) was used to calculate concentrations and purity. Samples used for RNA-seq were also analyzed using an Agilent 2100 Bioanalyzer (Agilent Genomics, Santa Clara, CA, USA). RNA-seq libraries were prepared following the manufacturer’s instructions from isolated total RNA using the Illumina TruSeq RNA Sample Preparation Kit (Illumina, San Diego, CA, USA). The libraries were sequenced using the manufacturer’s protocol of the Illumina GAIIx platform for 72 paired-end cycles. Sequences are publicly available through the Gene Expression Omnibus database under the accession number GSE123522. Contamination and adapter removal were carried out using in-house Perl scripts. Fastq sequences were filtered based on quality (FASTQ Quality Filter v0.0.6, Q 33, http://hannonlab.cshl.edu/fastx_toolkit/index.html) and mapped to the *A. thaliana* transcriptome (TAIR10) using Bowtie2 [74]. Gene expression was determined using RSEM v1.3 [75] and compared between the RNA-seq libraries using DEGseq v3.6 [76] and the FPKM data from RSEM. Only transcripts with a Log2 fold change of <−1 or >1 with a *p*-value < 0.05 were considered. DEGs identified by genome-wide transcriptomic analysis were analyzed and classified into gene ontology classes (GO) using the PANTHER Gene List Analysis tools and default parameters. The identification of commonly regulated DEGs was performed using the software FiRe ver. 2.2, as previously described [77]. The MapMan software was used to visualize the amplitudes of the changes in the expression of individual genes in diagrams of cellular processes, as previously described [36].

### 4.9. Quantitative Real Time RT-PCR

The pooled total RNA (1.0 µg) used for RNAseq analysis was retro-transcribed into cDNA according to the manufacturer’s indications using the SCRIPT cDNA Synthesis Kit (Jena Bioscience, Jena, Germany). qRT-PCR was performed in 96-well plates with the Applied Biosystems StepOne™ and StepOnePlus™ Real-Time PCR System (ThermoFisher Scientific, Waltham, MA, USA) using the SYBR Green Maxima SYBR Green/ROX qPCR Master Mix (2X) (ThermoFisher Scientific, Waltham, MA, USA). Three independent experiments were analyzed, each with three technical replicates. The qRT-PCR conditions were as follows: an initial 95 °C denaturation step for 5 min, followed by denaturation for 15 s at 95 °C, annealing for 30 s at 60 °C, and extension for 30 s at 72 °C for 45 cycles. Gene expression values were normalized using the mean expression of two genes: AT4G26410 (*RHIP1*) and AT1G72150 (*PATL1*), which were previously described as stable reference genes [44,78]. Normalized gene expression was determined using the comparative 2^−∆∆CT^ method, as previously described [79]. Primers for gene expression analysis have been previously described: for *PR1*, *PDF1.2* and *PR4* [43]; for *ZAT12* [37], *ICS1* [80] and *ACS6* [81].

### 4.10. Statistical Analysis

All results are reported as mean values (±SD) and were analyzed by an analysis of variance and compared by the Scott–Knott test (*p* < 0.05) using the software GraphPad Prism version 8.1.0 (2019, GraphPad Software, San Diego, CA, USA). All the data analyzed were obtained from three independent experiments.

## Data Availability

Sequences are publicly available through the Gene Expression Omnibus database under the accession number GSE123522.

## Supplementary Materials

The following are available online at https://www.mdpi.com/article/10.3390/ijms22094938/s1, Supplementary Table S1: GO enrichment analysis of differentially expressed genes commonly induced or repressed after Gd application (24 hpt) and during the interaction with *B. cinerea* (24 hpi), Supplementary Figure S1: Gd does not affect germination rates, fresh nor dry weight, Supplementary Figure S2: Gd does not modify plant cuticle permeability, Supplementary Figure S3: MapMan pathway analysis of differentially expressed genes of (A) 24 hpt with 0.2 gL^−1^ Gd and (B) afterwards infected with with *B. cinereal* (24 hpi) compared to Ca-treated samples, Supplementary Data 1: List of significantly differentially expressed genes 24 hpt, Supplementary Data 2: List of significantly differentially expressed genes 24 hpi.
