# Supplementary material for: Gadolinium Protects Arabidopsis thaliana against Botrytis cinerea through the Activation of JA/ET-Induced Defense Responses
Source: Int J Mol Sci. 2021 May 6;22(9):4938. doi: 10.3390/ijms22094938 (PMC8124739; doi:10.3390/ijms22094938)
Supplement: Supplementary file 1 [file ijms-22-04938-s001.zip › Figure legends Supplementary.pdf]

Supplementary Information Legends.

Supplementary Table 1. GO enrichment analysis of differentially expressed genes commonly induced or repressed after Gd application (24 hpt) and during the interaction with B. cinerea (24 hpi).

Supplementary Figure 1. Gd does not affect germination rates, fresh nor dry weight. (**A**). Mock-, Gd- or Ca-treated seedlings were transplanted to the soil and 30 days post transplanting the number of leaves were counted A. thaliana seeds were incubated during 1 hour with distilled sterile water (Mock), 0.2 gl-1 Ca or Gd and germinated under in-vitro conditions, 10 days post incubation germination rate (**B**), fresh (**C**) and dry weight (**D**) were measured. Bars represent mean values (± SD) of three independent experiments (n=30 for each experiment). Different letters above each bar represent statistically significant differences according to the Scott-Knott test (p<0.05).

Supplementary Figure 2. Gd does not modify plant cuticle permeability. 4-week-old A. thaliana plants were treated with 0.2 gl-1 Ca or Gd (24 hpt). (**A**) Leaves were placed in ethanol and the release of chlorophyll was followed over the indicated time points. The values represent mean of three independent samples (± SD). (**B**) A droplet of toluidine blue was placed on the leaf surface for 2 hours, then was rinsed away with water. The blue stain remains attached to the cell wall if the plant has a more permeable cuticle. (**C**) Leaves were bleached overnight in ethanol then stained with calcofluor white that binds to cellulose and viewed under UV light. Calcofluor staining to the leaf is indicative of a permeabilized cuticle. All the experiments were carried out three times with similar results, representative pictures are shown.

Supplementary Figure 3. MapMan pathway analysis of differentially expressed genes of (**A**) 24 hpt with 0.2 gl-1 Gd and (**B**) afterwards infected with with B. cinereal (24 hpi) compared to Ca-treated samples.

Supplementary Data 1. List of significantly differentially expressed genes 24 hpt. Genes names are from TAIR 10 annotation. Ca- and Gd-treated columns contain the FPKM values from RSEM analysis, from Ca- and Gd-treated A. thaliana samples, respectively. “Fold change” are normalized Log2 values. All the transcripts of this table have a p-value <0.001. The genes analyzed on Figure 7 are highlighted in yellow.

Supplementary Data 2. List of significantly differentially expressed genes 24 hpi. Genes names are from TAIR 10 annotation. Ca- and Gd-treated columns contain the FPKM values from RSEM analysis, from Ca- and Gd-treated A. thaliana and infected with B. cinerea samples, respectively. “Fold change” are normalized Log2 values. All the transcripts of this table have a p-value <0.001. The genes analyzed on Figure 7 are highlighted in yellow.
