# Supplementary figures and images for: Gadolinium Protects Arabidopsis thaliana against Botrytis cinerea through the Activation of JA/ET-Induced Defense Responses
Source: Int J Mol Sci. 2021 May 6;22(9):4938. doi: 10.3390/ijms22094938 (PMC8124739; doi:10.3390/ijms22094938)

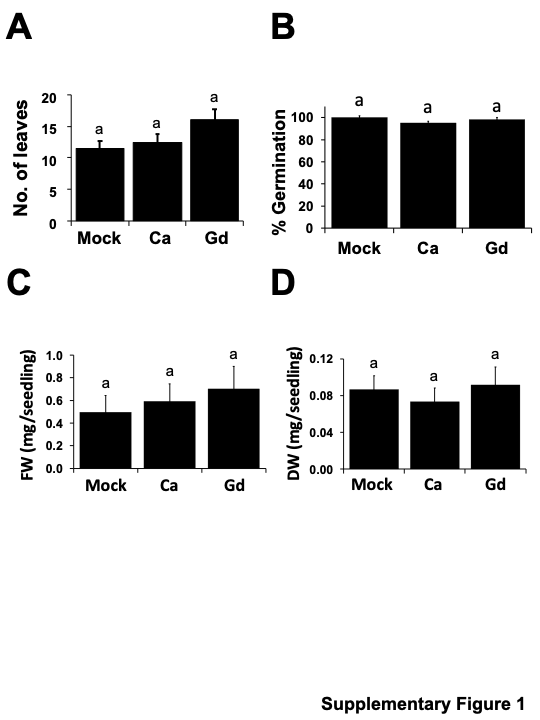

Supplement: Supplementary file 1 [file ijms-22-04938-s001.zip › Supplementary Figure 1.tiff]

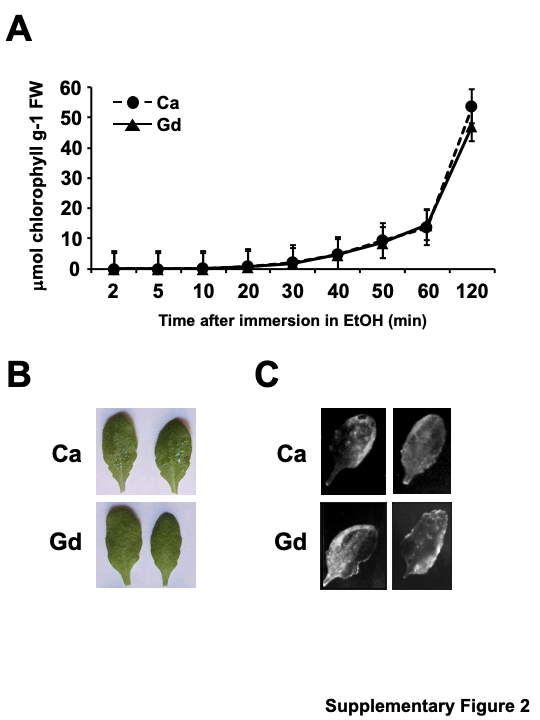

Supplement: Supplementary file 1 [file ijms-22-04938-s001.zip › Supplementary Figure 2.tiff]

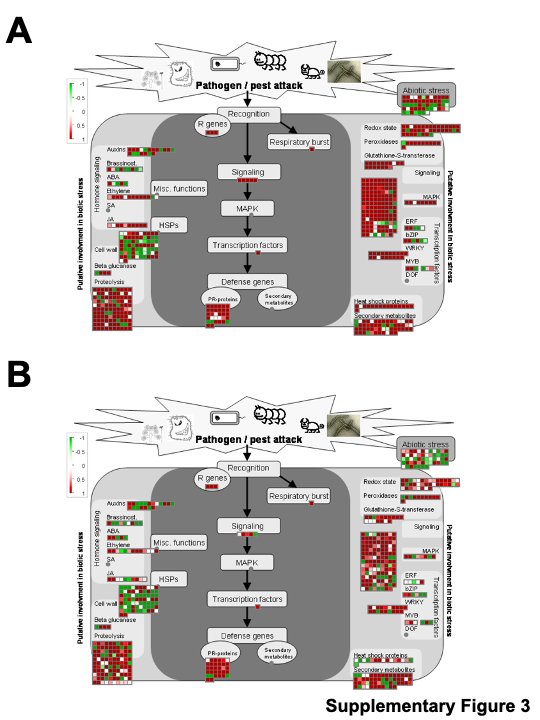

Supplement: Supplementary file 1 [file ijms-22-04938-s001.zip › Supplementary Figure 3.tiff]
